# Prevention of TGF-β-induced early liver fibrosis by a maleic acid derivative anti-oxidant through suppression of ROS, inflammation and hepatic stellate cells activation

**DOI:** 10.1371/journal.pone.0174008

**Published:** 2017-04-06

**Authors:** Kun-Lin Yang, Wen-Teng Chang, Ming-Yuan Hong, Kuo-Chen Hung, Chia-Chang Chuang

**Affiliations:** 1 Department of Animal Science, National Pei-men Senior Agricultural and Industrial Vocational School, Tainan, Taiwan; 2 Department of Biotechnology, National Kaohsiung Normal University, Kaohsiung, Taiwan; 3 Department of Biological Science and Technology, Chung Hwa University of Medical Technology, Tainan, Taiwan; 4 Department of Emergency Medicine, National Cheng Kung University Hospital, College of Medicine, National Cheng Kung University, Tainan, Taiwan; 5 Department of General Surgery, Yuan’s General Hospital, Kaohsiung, Taiwan; Saint Louis University, UNITED STATES

## Abstract

Current anti-fibrotic effect of antioxidants *in vivo* is disappointing due probably to the fact that once liver fibrogenesis is established it is too advanced to be reversed by anti-oxidation mechanism. We consider antioxidant may only act on the early phase of fibrogenesis. Thus, we had previously established an early liver fibrosis animal model using an inducible expression vector (pPK9a), which contains TGF-β gene and was hydro-dynamically transferred into mice to induce a transient liver fibrosis. TGF-β1 has been well documented to up-regulate the expression of α2(1) collagen (Col 1A2) gene in the liver via the reactive oxygen species (ROS); the process triggers inflammation, leading to hepatic stellate cells (HSC) activation and liver fibrogenesis. Using our animal model and ROS, cyclooxygenase-2 (Cox-2) and Col 1A2 promoter assays as screening targets, we report here that a maleic acid derivative isolated from the *Antrodia camphorata* mycelium strongly decreases ROS production, promoter activity of Cox-2 and Col 1A2, intracellular calcium, expression of alpha-smooth muscle actin (α-SMA), Smad4-p-Smad2/3 co-localization in cell nucleus and the DNA binding activity of Sp1. Our results suggest that the maleic acid derivative prevents liver fibrosis at an early phase both *in vitro* and *in vivo* through the inhibition of ROS, inflammation and the activation of HSC.

## Introduction

Advanced liver fibrosis represents a significant and severe healthcare problem and there are no effective drugs for therapy so far. Hence, prevention of progression of fibrogenesis and revival of endogenous repair activities at an early stage is an important matter for both current and future therapies. Accordingly, it becomes important to have a suitable fibrosis model for studying the initiating stage of fibrosis and finding therapeutic molecules for targeting in early fibrogenesis.

TGF-β1 is one of the powerful and widely distributed profibrogenic mediators and plays predominant role during organ fibrogenesis. In the liver, TGF-β1 transforms hepatic stellate cells (HSC) into myofibroblasts which are the main source of liver extracellular matrix (ECM). In response to TGF-β stimulation HSC induces up-regulated expressions of Col 1A2 and α-smooth muscle actin (α-SMA), the two indicators of fibrosis; the process is mediated by phosphorylation of Smad2 and Smad3 and subsequent nuclear translocation of a Smad containing complex. Moreover, it has also been documented that TGF-β1 up-regulates the expression of *Col 1A2* gene in the liver via ROS generation and calcium influx [1, 2]. The up-regulated Ca^2+^ influx is associated with activation of HSC and cell contraction [3]. The cascade process triggers inflammation, leading to HSC damages and liver fibrogenesis [4].

Hydrodynamics-based gene delivery is an efficient, simple and convenient transfection procedure for laboratory animals where a large-volume naked plasmid DNA is intravenously injected into the animal tail vein at a high-speed. The method allows the achievement of a high expression level of exogenous gene in the liver [5, 6]. Using this approach we have recently established a transient and early fibrosis animal model by using a pPk9a plasmid that contains TGF-β1 gene and was transferred via tail vein injection into a testing animal. TGF-β1 expression in this transgenic model was high and transient [5, 6].

**Fig 1:**
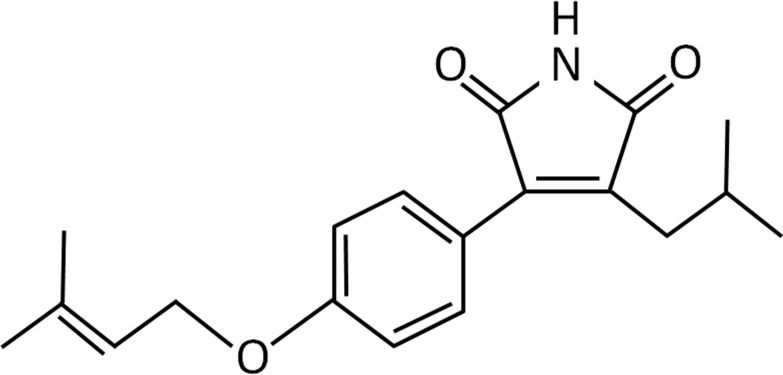
Structure of the compound 2 molecule (molecular formula C_19_H_23_NO_3_, m.w. = 313).

Various molecules designed to affect the TGF-β system have been successfully used for anti-fibrogenesis studies [7]. Blockage of ROS generation by HSC in response to TGF-β and alleviation of the downstream proteins is a strategy to inhibit liver fibrosis. We hence set out to screen small molecules isolated from some herbal medicines with the hope that they may antagonize fibrosis. In the process we used ROS, Col 1A2 and Cox-2 promoter assays as screening targets. Out of a panel of approximately 96 chemical compounds a small maleic acid derivative molecule isolated from the mycelia of *Antrodia camphorata* (designated here as compound 2) (Fig 1) was found to be a promising candidate molecule for inhibiting liver fibrogenesis. The fruiting body of *A*. *camphorata* is a well-known traditional herbal medicine in Taiwan, it has been shown to have antioxidant and anti-inflammatory effects [8–11]. The maleic acid derivative is attributed to be the main component in *A*. *camphorata* for the antioxidant and anti-inflammatory activities. Our finding may have interesting clinical implication.

## Materials and methods

### Experimental procedures

#### Cell line

HSC-T6 cells are immortalized and closely resembling primary stellate cells of rat liver. This cell line is a generous gift from Professor S. L. Friedman of the Mount Sinai School of Medicine (NY, USA) [12]. The cells were cultured in Dulbecco’s modified Eagle’s medium containing 10% fetal bovine serum at 37°C with 5% CO_2_ in a humidified incubator and medium was replaced every two days.

#### Mice

Mice of BALB/c strain were used as a transient fibrosis animal model for this study. All procedures of animal handling were approved by the Institutional Animal Care and Use Committee of the National Cheng Kung University. The mice (8 weeks old) were divided into seven groups (n = 21), fed *ad libitum* standard laboratory chow and water with or without 25 mM of ZnSO_4_ plus 5% sucrose. Compound 2 was dissolved in DMSO and given intraperitoneally (i.p.) at 30, 150 or 300 μg/kg. For hydrodynamic transfer of pPK9a vectors containing TGF-β1 gene into mice, the vectors were injected through tail vein simultaneously with ZnSO_4_ and compound 2 [13].

#### Chemical

Compound 2 was isolated and purified from *A*. *camphorata* mycelium by the Simpson Biotech Co. Ltd., Taiwan [14]. The compound is a maleic acid derivative with a formula shown in Fig 1.

#### cDNA constructs

A pPK9a vector containing TGF-β1 cDNA is a gift from Professor Paturu Kondaiah. TGF-β1 gene is triggered by the promoter of metallothionein gene, which is expressed in liver. As indicated by reference [15], both Cys^223^ and Cys^225^ in the TGF-β pro-peptide were converted into serine, resulting in the dissociation of the pro-peptide and *de novo* secretion of bioactive TGF-β1 (25 KD). The bioactive TGF-β1 significantly stimulates the activation of a 353-bp fragment of the *Col 1A2* gene promoter [16]. The -353/-1 fragment constructed into the pGL2-basic vector is a generous gift of Professor Maria Trojanowa. The promoter-reporter construct pxc918 containing -918/+49 COX-2 promoter fragment is also a gift from Dr. Wen-Chang Chang [17].

#### Measurement of [Ca^2+^]i in HSC-T6 cells

Observation of [Ca^2+^]i under the treatment of TGF-β and compound 2. Cells were first pretreated with compound 2 (30, 150 or 300 nM) for 8 h then loaded with the fluorescent calcium indicator fura-3/AM before the addition of TGF-β1 (2 ng/ml). Cell images were taken using a florescence microscope.

#### Measurement of ROS production from HSC-T6 cell

To analyze the effects of compound 2 on ROS production after TGF-β treatment, ROS was measured by chemiluminescence (CL) count. CL are highly sensitive and specific to the different kinds of ROS simultaneously, such as HO^·^, O_2_^·-^ and H_2_O_2_. The luminol-dependent CL have been used frequently for the detection of superoxide radical anions in various biological systems. HSC-T6 cells (1 × 10^6^ cells) were incubated at 37°C with different concentrations of compound 2 (0–300 ng/ml) and 2 ng/ml TGF-β for 4 hours. After treatment, HSC-T6 cells were collected and suspended in 0.2 ml PBS. The CL count was measured in a completely dark chamber of the Chemiluminescence Analyzing System. After determination of a 60-s background level, 0.5 ml of 25 mM luminol in PBS (pH 7.4) was injected into the sample. The CL was monitored continuously for an additional 240 s.

#### Dual-luciferase assay

HSC-T6 cells (1.5 × 10^5^) were cultured in each well of six-well plates. The calcium phosphate precipitation method was used for transient transfection [18]. The plasmids pRL-TK and p353 Lux (Col 1A2-luciferase) or pxc918 Lux (Cox-2- luciferase) were co-transfected into cells. The pRL-TK vector was used to normalize the transfection efficiency. After transfection for 12 h, the medium was changed and the cells were treated with various concentrations of compound 2 (30, 150, 300 nM) and TGF-β (2 ng/ml) for additional 24 h. The cells were washed in PBS for recovering cell lysate by scraping the cells in the presence of 1 × passive lysis buffer (Promega, Madison, WI). Firefly and *Renilla* luciferase activities were analyzed using the Dual-luciferase assay system (Promega) and measured by a Sirius luminometer (Berthold Detection System, Pforzheim, Germany).

#### Cellular immunofluorescence imaging

HSC-T6 (1×10^5^ cells/well) were seeded into six-well Lab-Tek II chamber glass slides (Nalge Nunc International, Naperville, IL) with complete medium. After 24-h culture, the medium was changed with fresh medium containing 0.1% FBS. Following treatment with 2 ng/ml of TGF-β1 and various concentrations of compound 2 (30, 150, 300 nM) for 8 h, cells were incubated on ice for 5 min, washed with ice-cold PBS, fixed with 100% methanol at -20°C, and washed twice at room temperature with PBS. The fixed cells were subjected to immunostaining and observed using a fluorescent or confocal microscopy [19, 20].

#### Hydrodynamics-based gene transfer

Ten mg of pPK9a plasmid was dissolved in 3.0 ml Ringer’s solution and injected *in bolus* into the mouse tail vein in a short duration of 5–7 s according to the hydrodynamics-based transfection protocol as described [5, 21, 22]. Drinking water contains 25 mM of ZnSO_4_ for gene-transferred animals to activate the metallothionein promoter and express TGF-β [13].

#### Histology and immunohistochemistry

Liver tissues of TGF-β gene-transferred mice were embedded in an optimal cutting temperature compound (Miles Inc., Elkhart, IN) and frozen in liquid nitrogen for following experiments. Five mm of cryosections were performed by using cryostats (Leica CM 1800, Nussloch, Germany). Histology and immunohistochemistry were performed as described previously [5]. For the detection of α-SMA, a mouse monoclonal antibody (Santa Cruz) was used. Signals were visualized by anti-mouse IgG, horseradish peroxidase labeled secondary antibody, and 3, 3'-diaminobenzidine substrate (Vector Laboratories, Burlingame, CA). All sections were viewed under a microscope (Leica Mikrosysteme Vertrieb GmbH, Bensheim, Germany).

#### Masson’s trichrome staining

Liver specimens from TGF-β gene-transferred mice were treated with 4% paraformaldehyde in PBS and dehydrated in a graded alcohol series. Following xylene treatment, the specimens were embedded in paraffin blocks and cut into 5 μm-thick sections that were stained with Masson’s trichrome as described [5, 23].

#### Immunoblotting

Liver tissues were homogenized in RIPA buffer as described [22, 24] and the supernatant was recovered as a whole-cell lysate. The proteins were analyzed on a 12% SDS-polyacrylamide gel electrophoresis. Anti-phospho-Smad 2/3 (Ser433/435- phosphorylated Smad2/3; p-Smad2/3), anti-Smad 2/3, anti-GAPDH (Santa Cruz), anti-Cox-2 antibody (Transduction Laboratories) and anti-α-SMA anti-body (Lab Vision Co.) were used as the primary antibodies. Secondary antibodies were conjugated with horseradish peroxidase (Bio-Rad Laboratories).

#### Gel electrophoretic mobility shift assay (EMSA)

The preparation of liver nuclear extracts and EMSA was based on the method described previously with minor modifications [20, 25].

#### Statistics

Results were expressed as mean ± SE. Statistical analysis was performed by unpaired Student's t test for pairwise comparisons. A p value <0.05 was regarded as statistically significant.

## Results

### Effects of compound 2 on intracellular ROS production

**Fig 2:**
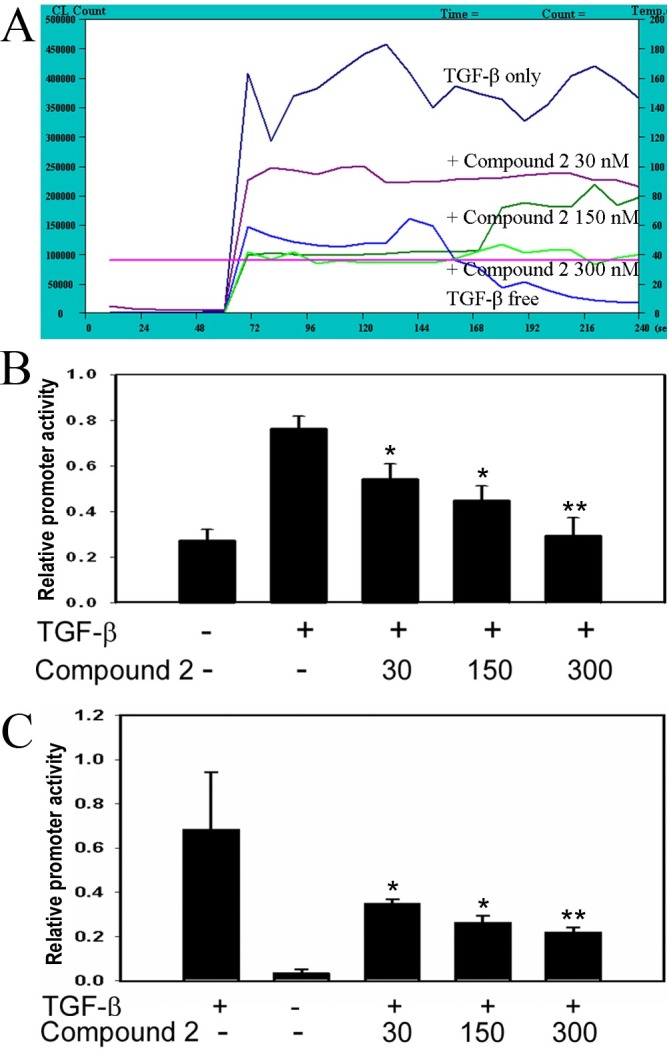
Reduction of ROS generation and promoter activity by compound 2 in TGF-β1-treated HSC-T6 cells. (A) Measurement of ROS under the treatment of TGF-β1 and compound 2. Cells incubated for 4 h in the presence of TGF-β1 and compound 2 were harvested for analyzing the oxidation state by adding 0.5 ml of 25 mM luminol. (B) & (C) Effects of compound 2 at various concentrations on the promoter activity of Col 1A2 (B) and Cox-2 (C) in the presence of TGF-β. The relative luciferase activity was expressed as ratio of firefly to Renilla luciferase activity. Values are represented as mean ± SD.; *, p<0.05, and **, p<0.01, unpaired *t* test, n = 4, compared with TGF-β only.

The intracellular oxidation state of the cell was analyzed by flow cytometry, when ROS were induced by TGF-β1 (2 ng/ml) in the presence or absence of compound 2. Pretreatment of the cells with 30–300 nM of compound 2 eliminated the ROS generation in a dose-dependent manner (Fig 2A). This result indicates that compound 2 may act as an antioxidant to block TGF-β-induced ROS.

### Effects of compound 2 on the promoter activities of Col 1A2 and Cox-2 genes

Transcription factors Sp1, AP-1, and Smad binding sites on the promoter region (-334 to -184) of *Col 1A2* have been implicated in TGF-β1 induced transcription in fibroblasts [26]. We used the fragment 353 bp of Col 1A2 and the fragment 918 bp of Cox-2 on promoter regions to investigate the effects of compound 2 on promoter activity [17]. Luciferase activities were assayed after cotransfection with pRL-TK and p353 Lux (Col 1A2-luciferase) or pxc918 (Cox-2-luciferase) in HSC-T6 cells, and then cells were cultured with compound 2 and TGF-β1 (2 ng/ml). HSC-T6 cells treated with compound 2 (30, 150, 300 nM) had a reduction of 40 to 70% luciferase activity with p353 Lux promoter (Fig 2B) and 30 to 50% reduction with pxc918 promoter (Fig 2C) in response to TGF-β1 stimulation. Compound 2 thus may simultaneously down regulate both fibrotic (Col 1A2) and inflammatory (Cox-2) processes *in vitro*.

### Compound 2 directly decreased the level of intracellular calcium ([Ca^2+^]i) in HSC-T6 cells

**Fig 3:**
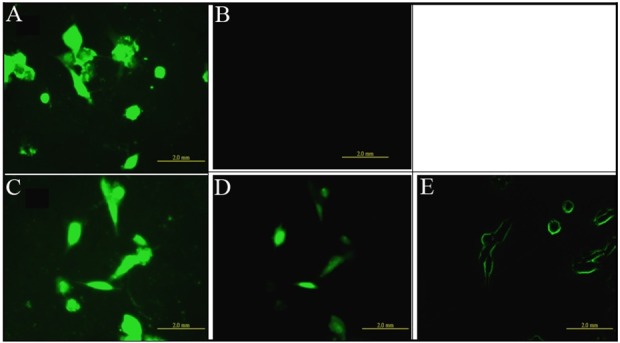
Effects of compound 2 on [Ca^2+^]_i_ in HSC-T6 cells. Observation of [Ca^2+^]_i_ under the treatment of TGF-β1 and compound 2. Cells were first pretreated with various concentrations of compound 2 (30, 150 or 300 nM) for 4 h then loaded with the fluorescent calcium indicator fura-3/AM before the addition of TGF-β1 (2ng/ml). (A) TGF-β only, (B) w/o TGF-β, (C) TGF-β + 30 nM compound 2, (D) TGF-β + 150 nM compound 2, and (E) TGF-β + 300 nM compound 2.

Generation of ROS has been reported to be a potential mechanism for alteration of intracellular calcium and up-regulation of ECM protein accumulation [2]. Therefore, we examined whether compound 2 could change the concentration of [Ca^2+^]_i_ in TGF-β treated HSC-T6 cells. After incubation with compound 2 at 30, 150, or 300 nM for 4 h, [Ca^2+^]_i_ was measured immediately upon addition of TGF-β1 to the cells (Fig 3). [Ca^2+^]_i_ compared with positive control were significantly reduced. We also used another calcium indicator fura-2/AM to measure real-time intracellular calcium concentration and found the same results (data not shown). The results imply that a trace amount of compound 2 is sufficient enough to block both [Ca^2+^]_i_.

### Effects of compound 2 on Smad4 and p-Smad2/3 in HSC-T6 cells

**Fig 4:**
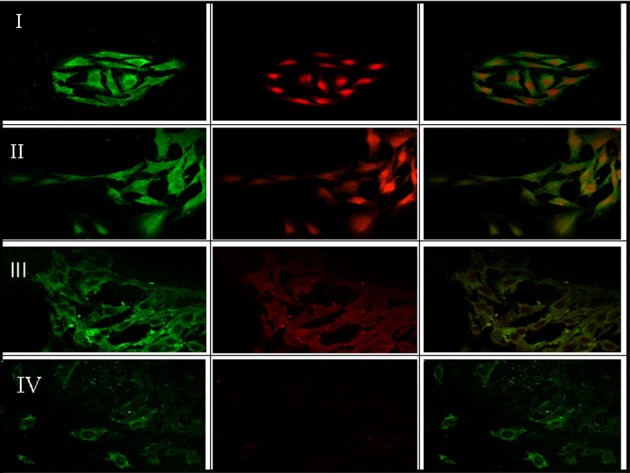
Effects of compound 2 on Smad4 and p-Smad2/3 localization in HSC-T6 cells. HSC-T6 cells were treated with TGF-β only (I), TGF-β + 30 nM compound 2 (II), TGF-β + 150 nM compound 2 (III), and TGF-β + 300 nM compound 2 (IV). Smad4 and p-Smad2/3 are showed in green and red colors, respectively.

Activation of HSC by TGF-β plays a key role in liver fibrosis at the early phase and the activated cells are accompanied with high accumulation of p-Smad2/3 and Smad4 in cell nucleus [2]. As shown in Fig 4, compound 2 alleviates both p-Smad2/3 and Smad4 accumulation in the nuclei in a dose-dependent manner and compound 2 also decreased levels of the p-Smad2/3 and Smad4 in cells. The result reveals that compound 2 also affects the activation of fibrosis-associated transcription factors.

### Effects of compound 2 on collagen expression in hydrodynamics-based fibrosis mice

**Fig 5:**
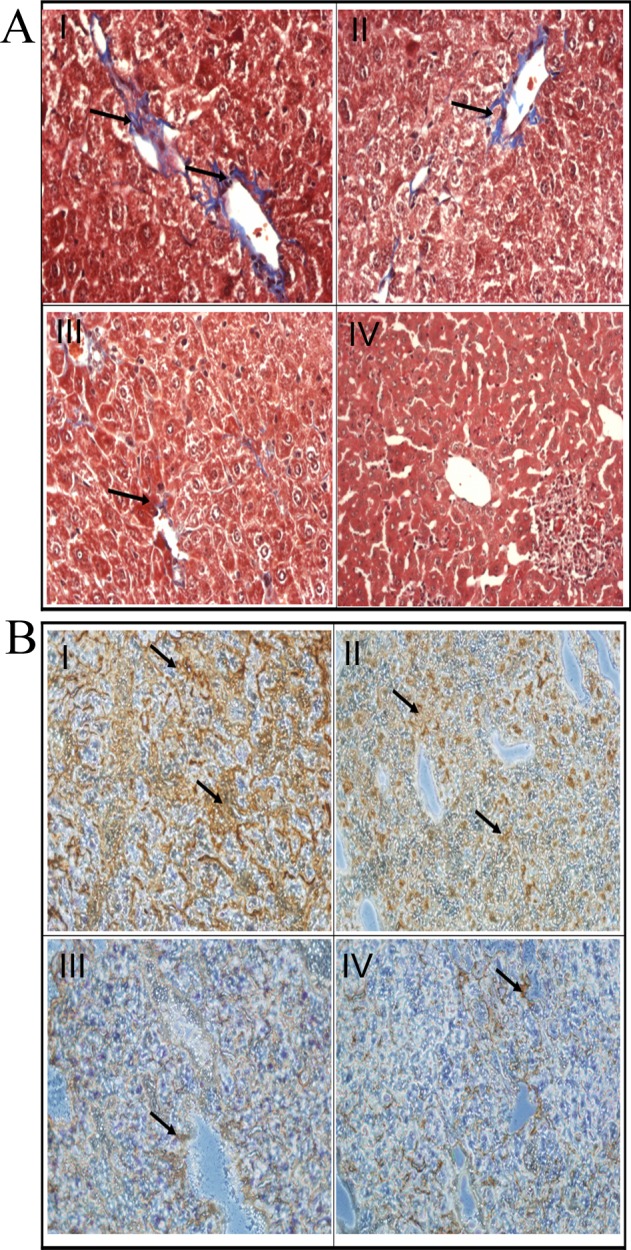
Effects of compound 2 on histopathology of liver fibrosis. Forty-eight hours after hydrodynamics-based injection of pPK9a, the mice were sacrificed and the liver sections were subjected to Masson’s trichrome staining (A) and immunohistochemistry (B). (A) Detection of ECM and collagen by Masson’s trichrome staining. The cytoplasm is red and collagen fiber is blue-green. The collagen signals were indicated by arrows. (B) Detection of α-SMA by immunohistochemistry. Dark brown granules represent α-SMA signals stained by α-SMA-specific antibody and were indicated by arrows. Representative liver sections of collagen (A) or α-SMA (B) from experimental I-VI groups: (I) Ringer’s solution + pPK9a + ZnSO_4_ 48 h, (II) Ringer’s solution + pPK9a + ZnSO_4_ + 30 μg/kg/day compound 2, (Ⅲ) Ringer’s solution + pPK9a + ZnSO_4_ +50 μg/kg/day compound 2, and (IV) Ringer’s solution + pPK9a + ZnSO_4_ + 300 μg/kg/day compound 2. Original magnification: I-IV, 200x.

We next assessed the effects of compound 2 *in vivo* using TGF-β transgenic mice. The degree of fibrosis was assessed by using the histopathological analysis of liver section under light microscope (Fig 5A). The Masson’s trichrome staining and immunohistochemistry showed that TGF-β1, induced by ZnSO_4_ treatment, triggered a hepatic injury 48 h after a hydrodynamics-based injection of pPK9a and compound 2 markedly inhibited accumulation of collagen and α-SMA expression in liver tissues (Fig 5A & 5B). These results indicate that compound 2 prevents TGF-β1-induced hepatic injury and liver fibrosis by a decrease of collagen accumulation.

### Western blot analysis for p-Smad2/3, α-SMA and Cox-2

**Fig 6:**
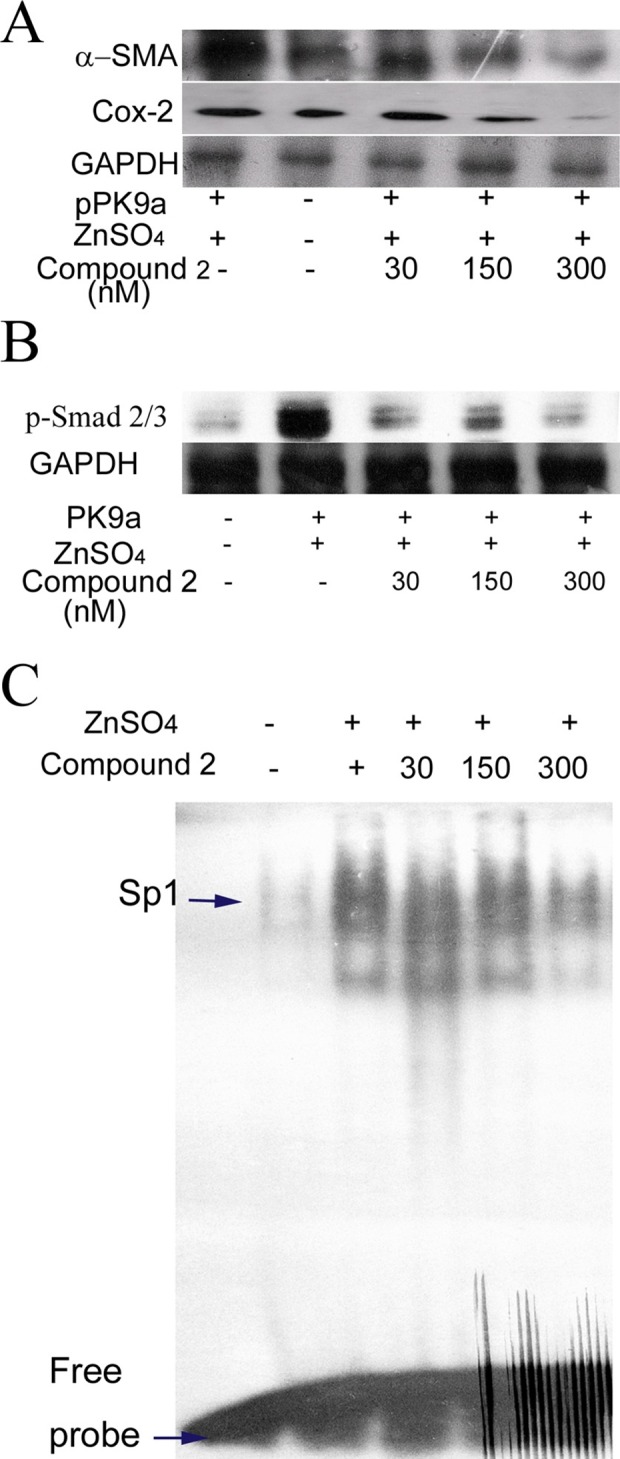
Effects of compound 2 on TGF-β1 downstream molecules in the livers of pPK9a-transferred mice. The total and nuclear proteins were extracted from the livers of pPK9a-transferred mice administrated with ZnSO_4_ water for 2 days. (A) & (B) Detection of protein levels of α-SMA, Cox-2, p-Smad, and GAPDH. Thirty μg of total proteins were subjected to Western blot and GAPDH is used as an internal control.

TGF-β plays a major role in fibrogenesis via the activation of HSC and accumulation of p-Smad2/3 in cell nucleus [26, 27]. Our *in vitro* study also showed the same finding (Fig 4). Therefore, we subsequently examined the levels of p-Smads and Cox-2 protein as well as HSC activation *in vivo*. We used immunohistostaining to measure the levels of p-Smads, Cox-2 and α-SMA in liver tissues of transgenic mice. α-SMA was used to detect the HSC activation. We found that compound 2 reduced the levels of p-Smad2/3, α-SMA and Cox-2 proteins in the liver of pPK9a-transferred mice in a dose-dependent manner (Fig 6A & 6B). The results indicate that compound 2 prevent fibrogenic effects of liver by decreasing p-Smad2/3, Cox-2 and HSC activation.

### Effects of compound 2 on Sp1 binding activity

TGF-β1 acts as a strong activator of ECM accumulation to induce the Col 1A2 gene expression by increasing the binding activity of a Sp1 and p-Smad2/3-Smad4 complex to Col 1A2 promoter element [27]. Since Sp1 is a critical mediator of Col 1A2 expression, we examined if Sp1 was induced in pPK9a-transferred mice treated with ZnSO_4_ and reversed by compound 2. The binding activity of Sp1 was performed with EMSA. Fig 6C shows that compound 2 indeed reduced the binding activity of Sp1 in liver in a dose-dependent manner. This result reveals that compound 2 not only affects the transcriptional activity of Smads but also Sp1 to regulate the expression of Col 1A2.

## Discussion

ROS intermediates act as signaling molecules of TGF-β in regulation of inflammatory and fibrotic responses [1, 2]. Therefore, first selective platform is used to ROS screen whether small molecules could act as an antioxidant to block TGF-β-induced ROS in HSC-T6 cells. The second and third selective platforms are the Col1A2 and Cox-2 promoter activities assays. We checked the molecules of TGF-β downstream signal pathway, such as Smads and Sp1, and we also checked the levels of collagen *in vivo*. In this study, we have demonstrated that compound 2, a maleic acid derivative isolated from the mycelium of *Antrodia camphorata*, can suppress the generations of ROS and [Ca^2+^]_i_, the activities of Col 1A2 and Cox-2 promoter, α-SMA expression and Smad protein localization in HSC-T6 cells induced by TGF-β (Figs 2–4). Liver fibrosis in mice was also greatly decreased when treated with compound 2 as represented by the Masson’s trichrome staining (Fig 5). Immunohistochemistry and RT-PCR showed that compound 2 can significantly inhibit collagen I production (Fig 5). Moreover, compound 2 decreased the TGF-β1 downstream signals, not only ROS and [Ca^2+^]_i_ *in vitro* (Figs 2 & 3) but also p-Smad 2/3 and Sp1 *in vivo* in hydrodynamics-based TGF-β gene transferred mice (Fig 6). We hence conclude that compound 2 acting as an oxygen free radical scavenger has anti-fibrotic effects and can suppress early phase of fibrosis.

**Fig 7:**
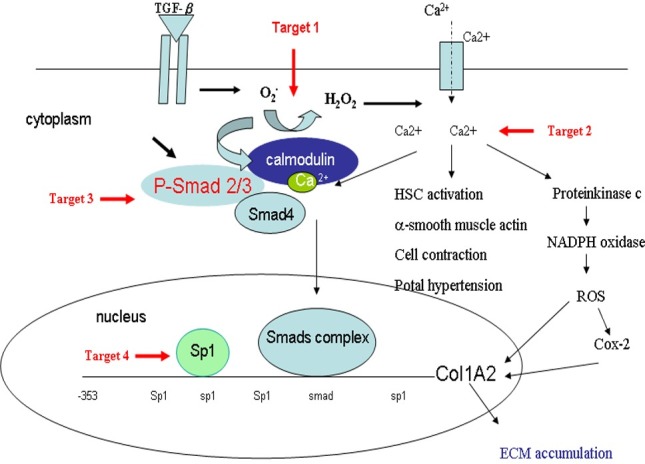
Schematic diagram of blocking targets of TGF-β1 signaling pathways in liver fibrosis.

The signaling relationship between TGF-β1 and ROS or Ca^2+^ has not been well elucidated in detail, although both ROS and Ca^2+^ have been documented to play important roles in the early phase of TGF-β1 stimulation [1]. It also has been reported that generation of ROS increases [Ca^2+^]_i_ and up-regulates ECM protein accumulation [2]. In this connection three points can be considered from our current work for possible blocking targets of TGF-β signaling pathways in liver fibrosis (Fig 7). First, our results demonstrate that a trace amount of compound 2, acting as an antioxidant, is sufficient enough to impede the generation of ROS (Fig 2) and the accumulation of [Ca^2+^]_i_ (Fig 3). The decrease of [Ca^2+^]_i_ interferes both the activation of HSC, in which α-SMA expression was reduced (Figs 3, 5 and 6), and the phosphorylation of Smad 2/3 (Fig 6). Secondly, the activation of protein kinase C is also able to induce Cox-2, which although is not addressed in our study but its downstream effecter Cox-2 was observed to be reduced in our study (Fig 6). And lastly, EMSA showed that compound 2 reduced the binding of Sp1 in the liver tissue in a dose-dependent manner, providing direct evidence that compound 2 down regulates the expression of Col 1A2 gene both *in vivo* and *in vitro* and therefore retards liver fibrosis induced by TGF-β1.

Our hydrodynamically transgenic mice procedure provides an animal model for studying early and initial fibrosis and the effects of antioxidants which may reverse fibrosis [5, 22]. Using this three selective platform and early liver fibrosis mice model, we had found two small molecules which are maleic acid derivative and retinoid acid derivative can inhibit fibrogenesis through the antioxidant and anti-inflammatory effects [22] And we had found two small molecules presents closely data in vitro and in vivo, it may cause by two small molecule through the same of tree selective platform. The phenomenon may seem to non-steroid anti-immflamentory drugs althought the drug different but function also similar. Our findings are important in that we present an interesting chemical and demonstrate its effects on the cascade molecular fibrogenetic events following ROS scavenging by compound 2 both *in vitro* and *in vivo*. Taken together our results might provide a new direction for developing therapeutic medicine for the prevention of early liver fibrosis.

## Supporting information

S1 File APPROVAL of NCKUH_IACUC[20170307]. (PDF)
