# Supplementary material for: Prevention of TGF-β-induced early liver fibrosis by a maleic acid derivative anti-oxidant through suppression of ROS, inflammation and hepatic stellate cells activation
Source: PLoS One. 2017 Apr 6;12(4):e0174008. doi: 10.1371/journal.pone.0174008 (PMC5383026; doi:10.1371/journal.pone.0174008)
Supplement: S1 File — (PDF) [file pone.0174008.s001.pdf]

收件編號:

國立成功大學
實驗動物照護與使用委員會審查同意書
Affidavit of Approval of Animal Use Protocol
National Cheng Kung University

同意書編號(IACUC Approval No)：930155

計畫主持人: 莊佳璋　　職　稱: 助理教授

單　位: 急診學科　　飼養地點: 成大動物中心

實驗地點: 成大動物中心

計 畫 名 稱: 建立抗肝臟纖維化活性成分的篩選平台

本「動物實驗計畫書」業經實驗動物照護與使用委員會■實質 □形式審查通過。本計畫預定飼養應用之動物如下：

動物種類(品系)	動物總數/計畫年數	飼養及應用期間(西元)
C57BC/6	100	2004/11/1~2005/10/31

The animal use protocol listed below has been reviewed and approved by the Institutional Animal Care and Use Committee (IACUC).

Protocol Title: Prevention of TGF-induced early liver fibrosis by a maleic acid derivative anti-oxidant through suppression of ROS, inflammation and hepatic stellate cells activation

IACUC Approval No：930155

Period of Protocol: Valid From: 11/1/2004 To: 10/31/2005

Principle Investigator (PI): Chuang-Chia Chang

實驗動物照護與使用委員會召集人：醫學院院長張俊彥(印)　日期：106. 3. 07 【Re-submitted】

IACUC Chairman: [illegible]ang-Yang Chang　Date: 106. 3. 07 【Re-submitted】

（重要文件，請勿遺失）

IACUC NCKU 2017.3.7 補發 收件章
